# Intensive blood pressure treatment in coronary artery disease: implications from the Systolic Blood Pressure Intervention Trial (SPRINT)

**DOI:** 10.1038/s41371-021-00494-8

**Published:** 2021-02-15

**Authors:** Jiabin Zang, Jianwen Liang, Xiaodong Zhuang, Shaozhao Zhang, Xinxue Liao, Guifu Wu

**Affiliations:** 1 Department of Cardiology, The Third Affiliated Hospital of Sun Yat-sen University, Guangzhou, Guangdong China; 2 Department of Cardiology, The Eighth Affiliated Hospital of Sun Yat-sen University, Shenzhen, Guangdong China; 3 Guangdong Innovative Engineering and Technology Research Center for Assisted Circulation, Shenzhen, Guangdong China; 4 Department of Cardiology, The First Affiliated Hospital of Sun Yat-sen University, Guangzhou, Guangdong China; 5 NHC Key Laboratory on Assisted Circulation, Sun Yat-sen University, Guangzhou, Guangdong China

**Keywords:** Hypertension, Vascular diseases

## Abstract

To investigate the optimal blood pressure (BP) in patients with coronary artery disease (CAD), we conducted subgroup analysis using SPRINT data. The study sample included 1206 participants with CAD (of whom 692 underwent coronary revascularization) and 8127 participants without CAD. Participants were randomized into two groups (systolic BP target of 140 mm Hg vs. 120 mm Hg). The primary outcome was a composite of cardiovascular events. After a median follow-up of 3.9 years, the hazard ratios (HRs) for the primary outcome were 0.65 (95% confidence interval (CI) 0.53–0.79) and 1.05 (95% CI 0.76–1.46) among those in the non-CAD and CAD subgroups, respectively (*P* value for interaction 0.02). Intensive BP treatment was a protective factor for all-cause death (HR 0.60, 95% CI 0.37–0.96) in the CAD subgroup, compared with standard BP treatment. The HRs (95% CI) for stroke were 3.57 (1.17–10.85) and 1.03 (0.29–3.62) among those in the coronary revascularization and non-revascularization subgroups, respectively (*P* value for interaction 0.13). For safety events, intensive BP treatment increased the risk of hypotension (HR 2.00, 95% CI 1.06–3.79) and electrolyte abnormalities (HR 2.38, 95% CI 1.25–4.56) in the CAD subgroup, while the risk of serious adverse events did not increase (HR 1.03, 95% CI 0.88–1.20). These results suggest that positive benefits from intensive BP treatment might be attenuated in patients with CAD who are under better secondary prevention. The risk of stroke might increase at the systolic BP target of 120 mm Hg in case of coronary revascularization, although the confidence interval was wide.

## Introduction

Large-scale prospective studies have demonstrated that elevated blood pressure (BP) is associated with coronary artery disease (CAD) [1–4]. The prevalence of hypertension ranges from 30 to 70% in individuals with pre-existing CAD [5], and a previous study demonstrated that a 20 mm Hg rise in systolic blood pressure (SBP) or a 10 mm Hg rise in diastolic blood pressure (DBP) results in a twofold increase in the risk of mortality among patients with ischemic heart disease aged 40–69 years [6]. Meanwhile, a reduction in SBP of 5 mm Hg can decrease the risk of death from cardiovascular disease (CVD) by 9% [7]. Currently, few clinical trials are primarily designed to evaluate optimal BP targets in patients with CAD. Therefore, there are no standardized BP targets for patients with CAD, and current clinical practice is largely based on expert consensus with scant clinical trial evidence. Recent practice guidelines recommend a BP target of less than 130/80 mm Hg in individuals with stable ischemic heart disease (SIHD), although it was acknowledged that this recommendation was supported by limited data [8]. Nevertheless, evidence suggests that adopting a “lower is better” approach for BP is far from ideal and that the BP targets vary depending on patient characteristics [9–12].

In hypertensive patients with CAD, atherosclerotic lesions and arterial stiffness tend to be more severe, resulting in a lower DBP and increased pulse pressure. Patients who have undergone coronary revascularization seem to be more tolerant of lower DBP than those who had not, which may be partly explained by improved myocardial perfusion [13]. When performed using the proper revascularization strategy for appropriate patients, coronary revascularization can offer survival benefits in CAD; however, there is a need for further research to define the optimal BP target and therapeutic benefit of intensive BP treatment in this population.

The Systolic Blood Pressure Intervention Trial (SPRINT) examined the effect of intensive BP treatment (SBP target <120 mm Hg) in hypertensive patients with high cardiovascular risk [14]. Using data from the SPRINT trial, we conducted a post-hoc analysis to investigate the optimal BP in CAD populations and in a subset of CAD patients who had undergone revascularization.

## Methods

### Data source and study population

Data were collected from the SPRINT study, which is a National Heart, Lung, and Blood Institute (NHLBI)-sponsored trial. SPRINT was a randomized, controlled, multicenter open-label trial that evaluated the effects of standard (SBP target <140 mm Hg) versus intensive (SBP target <120 mm Hg) BP treatment among 9361 adults with hypertension (SBP of 130–180 mm Hg). Patients with diabetes mellitus, prior stroke, congestive heart failure, and advanced chronic kidney disease were excluded. In this study, patients diagnosed with CAD by a physician were randomized to either a standard or intensive group. Patients were followed up monthly for the first 3 months and every 3 months thereafter until 5 years or closeout. Coronary revascularization was defined by self-report of a history of percutaneous coronary intervention or coronary artery bypass grafting. The detailed inclusion and exclusion criteria are presented in the SPRINT study [14].

### Interventions and measurements

BP measurements were taken with the participants in a seated position using the same automatic device in an unattended office. The mean of three measurements was included in the analysis. During a median follow-up of 3.9 years, BP-lowering medications were adjusted to an SBP target of 135–139 mm Hg in the standard group and to less than 120 mm Hg in the intensive group.

### Clinical outcomes and safety events

Composite events of myocardial infarction, acute coronary syndromes, heart failure, stroke, and cardiovascular death were defined as the primary outcomes. Secondary outcomes included all-cause death and the various elements of the composite primary outcome.

In this study, the following conditions were recorded as safety events: hypotension, syncope, electrolyte abnormality, injurious fall, acute kidney injury, and bradycardia. Serious adverse events (SAEs) were defined as fatal events that first caused significant dysfunction and required medical intervention or hospitalization.

### Statistical analysis

Standard descriptive statistics were applied to baseline characteristics of CAD status and coronary revascularization status. Further, we compared the baseline characteristics by BP treatment arms among patients with CAD.

We calculated mean SBP and DBP every 3 months and the mean number of BP-lowering medications every 6 months using all values from the two BP treatment groups during follow-up. These results were summarized in a line graph. Kaplan–Meier survival curves were plotted, and clinical outcomes and safety event rates were computed using Cox proportional hazards regression adjusted for covariates, including demographic characteristics (age and gender), health habits (smoking status), and health condition (body mass index; levels of fasting plasma glucose, lipoprotein cholesterol, and triglycerides; estimated glomerular filtration rate; and use of BP-lowering medications). In addition, we conducted landmark analysis to compare primary outcomes between groups. Furthermore, subgroup analysis was used to assess the effects of CAD status and coronary revascularization status on BP treatment strategies, and these data were presented in a forest plot. All tests were two-sided, and the significance level was set at *P* < 0.05. Statistical analyses were performed with SPSS software, version 25 (Chicago, IL, USA) and R 3.6.3 (http://www.R-project.org).

## Results

### Study cohort and population characteristics

Of the 9361 participants in the SPRINT study, there were 1206 participants with CAD (of whom 692 underwent coronary revascularization) and 8127 participants without CAD at baseline (Supplementary Fig. 1). There were significant differences in baseline characteristics between participants with and without CAD (Supplementary Table 1). The baseline characteristics of participants with and without coronary revascularization are shown in Supplementary Table 2.

**Fig. 1:**
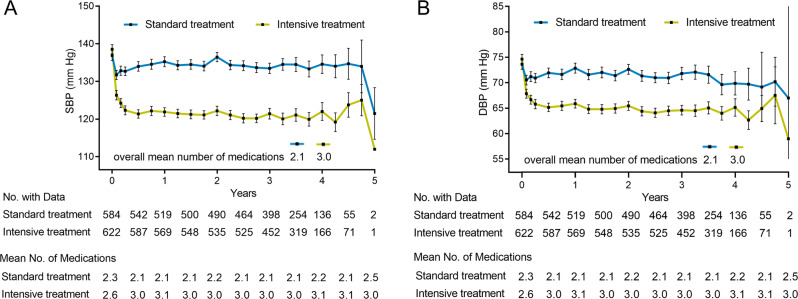
Blood pressure for participants with CAD through the 5-years follow-up visit. Mean follow-up SBP (**A**) and DBP (**B**) in the two BP treatment groups in participants with CAD. Mean number of BP-lowering medications are noted every 6 months during follow-up. Bars represent 95% CI. SBP systolic blood pressure, DBP diastolic blood pressure, CAD coronary artery disease, CI confidence interval.

The baseline characteristics of participants with CAD were comparable (*P* > 0.05) between the standard and intensive groups (Table 1). At baseline, the mean BP was 136.9 ± 16.2/73.6 ± 12.0 mm Hg in the standard group and 138.6 ± 15.5/74.7 ± 12.1 mm Hg in the intensive group. In both groups, the mean BP levels were controlled to within the target range (133.7 ± 3.0/71.0 ± 1.4 mm Hg vs. 120.9 ± 2.5/64.6 ± 1.6 mm Hg) during follow-up. The mean number of BP-lowering medications in the standard and intensive groups was 2.1 and 3.0, respectively (Fig. 1). The BP-lowering medications of different groups are summarized in Supplementary Tables 3 and 4.

**Table 1:** Baseline characteristics of CAD participants by BP treatment arm.

Characteristics	Total	Standard BP treatment	Intensive BP treatment	*P* value
*N*	1206	584	622	
Age, years	70.0 ± 9.1	69.7 ± 9.3	70.1 ± 9.0	0.38
Female, *n* (%)	246 (20.4)	132 (22.6)	114 (18.3)	0.07
Black race, *n* (%)	220 (18.2)	113 (19.3)	107 (17.2)	0.34
Body mass index, kg/m^2^	29.5 ± 5.4	29.7 ± 5.4	29.4 ± 5.3	0.38
Systolic blood pressure, mm Hg	137.8 ± 15.7	136.9 ± 16.2	138.6 ± 15.5	0.07
Diastolic blood pressure, mm Hg	74.1 ± 12.1	73.6 ± 12.0	74.7 ± 12.1	0.14
Heart rate, bpm	62.7 ± 11.1	63.2 ± 11.7	62.6 ± 10.7	0.34
Chronic kidney disease, *n* (%)	442 (36.7)	216 (37.0)	226 (36.3)	0.81
Smoking status, *n* (%)				0.67
Never smoked	401 (33.3)	188 (32.2)	213 (34.2)	
Former smoker	647 (53.6)	321 (55.0)	326 (52.4)	
Current smoker	158 (13.1)	75 (12.8)	83 (13.3)	
Total cholesterol, mg/dl	165.9 ± 39.2	167.4 ± 40.0	165.5 ± 40.2	0.42
LDL-C, mg/dl	92.4 ± 33.7	92.8 ± 33.2	91.8 ± 34.1	0.62
HDL-C, mg/dl	48.8 ± 12.2	48.7 ± 12.3	48.9 ± 12.3	0.71
Triglycerides, mg/dl	123.8 ± 62.3	128.9 ± 75.4	126.5 ± 91.8	0.61
Fasting plasma glucose, mg/dl	100.5 ± 13.5	100.1 ± 13.0	100.9 ± 14.0	0.27
eGFR, mL/min/1.73 m^2^	67.2 ± 19.8	67.1 ± 19.7	67.8 ± 20.0	0.55
Creatinine, mg/dl	1.2 ± 0.3	1.1 ± 0.3	1.1 ± 0.4	0.95
Serum sodium, mmol/l	140.2 ± 2.5	140.1 ± 2.5	140.2 ± 2.6	0.64
Serum potassium, mmol/l	4.3 ± 0.4	4.3 ± 0.4	4.3 ± 0.4	0.08
Statin use, *n* (%)	946 (78.4)	457 (78.3)	489 (79.6)	0.56
Aspirin use, *n* (%)	1027 (85.2)	488 (83.6)	539 (86.7)	0.13
Antihypertensive agents, *n* (%)				0.34
1	246 (20.4)	111 (19.0)	135 (21.7)	
2	472 (39.1)	227 (38.9)	245 (39.4)	
3	333 (27.6)	171 (29.3)	162 (26.0)	
4	97 (8.0)	47 (8.0)	50 (8.0)	

Values are mean ± SD or number (%).

*CAD* coronary artery disease, *LDL-C* low-density lipoprotein cholesterol, *HDL-C* high-density lipoprotein cholesterol, *eGFR* estimated glomerular filtration rate.

### Subgroup analysis by CAD status

**Fig. 2:**
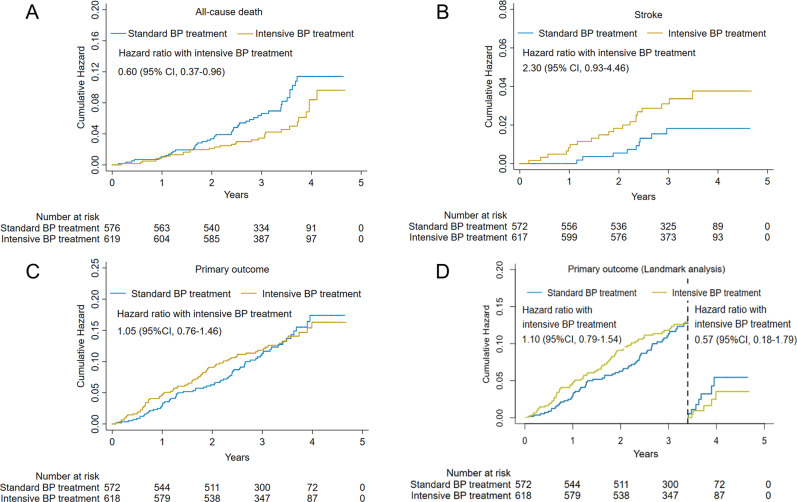
Cumulative incidence for clinical outcomes in participants with CAD, comparing treatment assignments. Cumulative incidence of the all-cause death (**A**), stroke (**B**), primary outcome (**C**), and the primary outcome with landmark analysis (**D**) according to standard and intensive BP treatments in participants with CAD. BP blood pressure, CAD coronary artery disease, HR hazard ratio, CI confidence interval.

After a median follow-up of 3.9 years (interquartile range 3.4–4.4 years), primary composite events were documented in 150 participants with CAD (70 in the standard group and 80 in the intensive group) (Table 2). Intensive BP treatment was associated with a lower risk of all-cause death (adjusted hazard ratio [HR] 0.60, 95% confidence interval [CI] 0.37–0.96) and a trend for an increased risk of stroke (adjusted HR 2.08, 95% CI 0.94–4.58), compared with standard BP treatment (Fig. 2A, B). The primary outcome, as well as most secondary outcomes, were similar in the two groups (Table 2, Fig. 2C). The landmark analysis showed that the HRs for the primary outcome were 1.10 (95% CI 0.79–1.54) and 0.57 (95% CI 0.18–1.79) within and after the first 3.4 years, respectively (Fig. 2D).

**Table 2:** Primary and second outcomes in CAD participants by BP treatment arm.

Outcome	Standard BP treatment	Intensive BP treatment	Unadjusted model		Adjusted model^a^	
			HR (95% CI)	*P* value	HR (95% CI)	*P* value
*N*	584	622				
Primary outcome	70 (12.0)	80 (12.9)	1.04 (0.76–1.44)	0.80	1.05 (0.76–1.46)	0.75
Secondary outcomes						
Myocardial infarction	28 (4.8)	32 (5.1)	1.03 (0.62–1.72)	0.90	1.05 (0.62–1.75)	0.87
ACS	16 (2.7)	21 (3.4)	1.20 (0.63–2.31)	0.58	1.22 (0.64–2.35)	0.55
Stroke	9 (1.5)	20 (3.2)	2.03 (0.93–4.46)	0.08	2.08 (0.94–4.58)	0.07
Heart failure	23 (3.9)	16 (2.6)	0.62 (0.33–1.18)	0.15	0.61 (0.32–1.17)	0.14
CVD death	15 (2.6)	13 (2.1)	0.77 (0.37–1.62)	0.49	0.75 (0.35–1.63)	0.47
All-cause death	45 (7.7)	31 (5.0)	0.62 (0.39–0.98)	0.04	0.60 (0.37–0.96)	0.03

Values are presented as number (%) or HR (95% CI).

*CAD* coronary artery disease, *ACS* acute coronary syndrome, *CVD* cardiovascular disease, *HR* hazard ratio, *CI* confidence interval.

^a^Adjusted model: age, gender, smoking status, body mass index, triglycerides, low-density lipoprotein cholesterol, high-density lipoprotein cholesterol, fasting plasma glucose, estimated glomerular filtration rate, and use of antihypertensive agents.

**Fig. 3:**
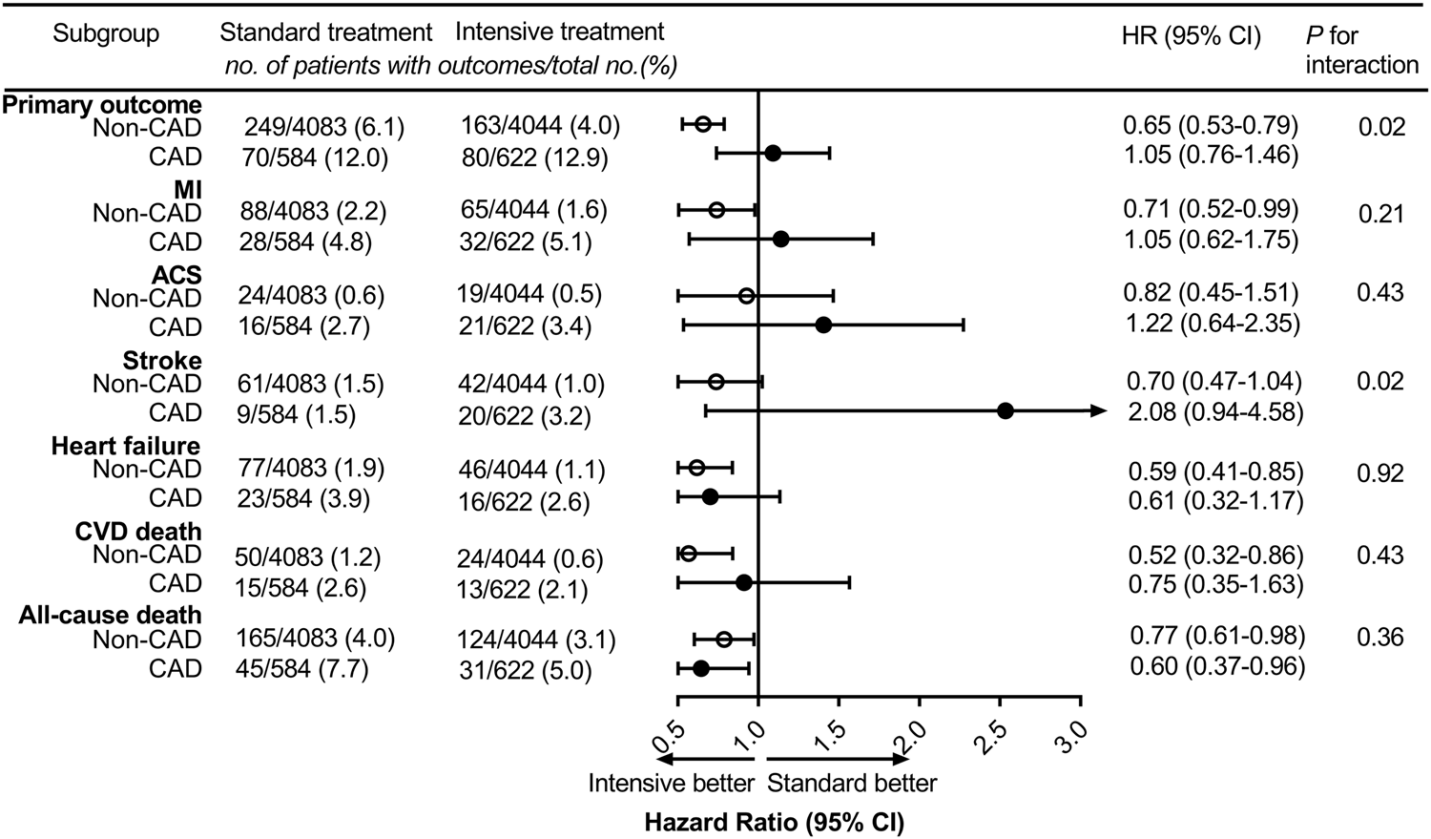
Test for interaction between BP treatment assignment and baseline CAD status. BP blood pressure, CAD coronary artery disease, MI myocardial infarction, ACS acute coronary syndrome, CVD cardiovascular disease, HR hazard ratio, CI confidence interval.

The SPRINT study showed that intensive BP treatment resulted in a low rate of major cardiovascular events, but we found no benefits on the primary outcome in participants with pre-existing CAD. Therefore, we further conducted subgroup analysis to verify whether CAD status plays a key role in the effects of BP treatment on clinical outcomes (Fig. 3). The results showed that the HRs for the primary outcome were 0.65 (95% CI 0.53–0.79) and 1.05 (95% CI 0.76–1.46) among those in the non-CAD and CAD subgroup, respectively (*P* value for interaction 0.02). For participants without CAD, intensive BP treatment significantly decreased the risk of myocardial infarction, heart failure, CVD death, and all-cause death, compared with standard BP treatment.

### Subgroup analysis by coronary revascularization status

**Fig. 4:**
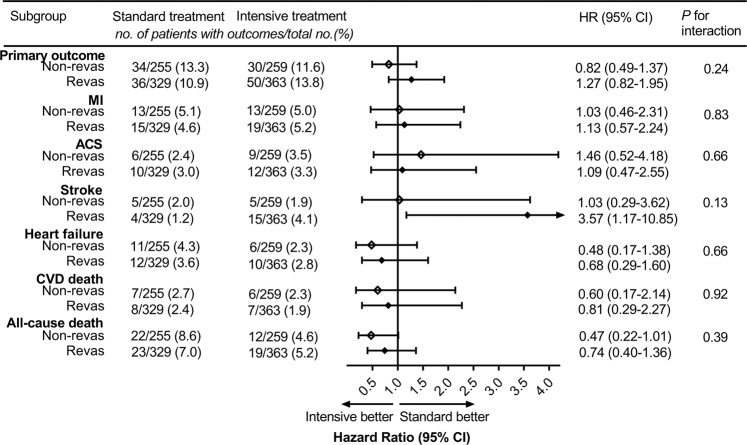
Test for interaction between BP treatment assignment and baseline coronary revascularization status in CAD participants. BP blood pressure, CAD coronary artery disease, Revas coronary revascularization, MI myocardial infarction, ACS acute coronary syndrome, CVD cardiovascular disease, HR hazard ratio, CI confidence interval.

In addition, participants with CAD were categorized by coronary revascularization status. The HRs for primary outcome were 1.27 (95% CI 0.82–1.95) and 0.82 (95% CI 0.49–1.37) in CAD participants with and without coronary revascularization, respectively (*P* value for interaction 0.24). Intensive BP treatment appeared to reduce the risk of all-cause death (HR 0.47, 95% CI 0.22–1.01) in participants without coronary revascularization, and there was no increase in the risk of stroke (HR 1.03, 95% CI 0.29–3.62). However, in the subgroup of participants with coronary revascularization, intensive BP treatment had no benefit on all-cause death (HR 0.74, 95% CI 0.40–1.36) but increased the risk of stroke (HR 3.57, 95% CI 1.17–10.85) (Fig. 4).

### Safety events in participants with CAD

Information regarding safety events in participants with CAD is summarized in Table 3. The incidence of total SAEs in the intensive group (54.7%, 340/622) was similar to that in the standard group (53.1%, 310/584). Intensive BP treatment increased the risk of hypotension (HR 2.00, 95% CI 1.06–3.79) and electrolyte abnormalities (HR 2.38, 95% CI 1.25–4.56). There was no increased risk of other safety events including syncope, injurious fall, acute kidney injury, and bradycardia in participants with intensive BP treatment, compared with standard BP treatment.

**Table 3:** Safety events in CAD participants by BP treatment arm.

Safety events^a^	Standard BP treatment	Intensive BP treatment	HR (95% CI)	*P* value
*N*	584	622		
Hypotension	14 (2.4)	30 (4.8)	2.00 (1.06–3.79)	0.03
Syncope	18 (3.1)	15 (2.4)	0.73 (0.37–1.47)	0.38
Electrolyte abnormality	13 (2.2)	33 (5.3)	2.38 (1.25–4.56)	0.01
Injurious fall	14 (2.4)	19 (3.1)	1.21 (0.60–2.43)	0.59
Acute kidney injury	25 (4.3)	38 (6.1)	1.39 (0.82–2.33)	0.22
Bradycardia	21 (3.6)	27 (4.3)	1.12 (0.63–1.98)	0.71
SAEs^b^	310 (53.1)	340 (54.7)	1.03 (0.88–1.20)	0.73

Values are presented as number (%) or HR (95% CI).

*CAD* coronary artery disease, *BP* blood pressure, *HR* hazard ratio, *CI* confidence interval.

^a^Safety events, including hypotension, syncope, electrolyte abnormality, injurious fall, acute kidney injury, and bradycardia.

^b^SAEs indicate serious adverse events, that resulted in significant dysfunction and required medical or hospitalization to surgical intervention.

## Discussion

Our results indicate that CAD and coronary revascularization status influence the effect of BP treatment on clinical outcomes. Intensive BP treatment decreased the risk of major cardiovascular events in participants without CAD, but not in those with CAD. For CAD participants, intensive BP treatment is associated with a reduced risk for all-cause death but does not affect other clinical outcomes, compared with standard BP treatment. The cardiovascular benefits from intensive BP treatment were further attenuated and the risk of stroke might increase in patients with CAD and a history of coronary revascularization.

The results of this study add to the complex evidence for the interaction and association of BP control with CAD status and coronary revascularization. We found a lower risk of clinical outcomes with an SBP target of 120 mm Hg among hypertensive patients without CAD. Attar et al. performed a further analysis of SPRINT participants categorized based on the baseline 10-year Framingham risk, and their results indicated that intensive BP treatment is beneficial for primary prevention of CVD and mortality in patients with high risk (above 10%) [15]. In contrast, another clinical trial (ONgoing Telmisartan Alone and in combination with Ramipril Global Endpoint Trial, ONTARGET) found that reducing SBP below 130 mm Hg failed to reduce cardiovascular mortality and that the optimal SBP was 135–145 mm Hg in patients with high cardiovascular risk [16].

The optimal BP targets in hypertensive patients with CAD remain controversial as few randomized clinical trials have directly evaluated this important clinical question. There are several existing studies nested with CAD populations, but these did not examine specific SBP targets. The INternational VErapamil SR Trandolapril STudy (INVEST) conducted by Pepine et al. evaluated 22,576 hypertensive CAD patients aged 50 years or older and found the verapamil-trandolapril-based strategy was as clinically effective as the atenolol-hydrochlorothiazide-based strategy, suggesting that reducing SBP is a far more important clinical consideration than the choice of antihypertensive drug class [17]. A post-hoc analysis of INVEST evaluated 8354 participants ≥60 years of age with hypertension and CAD and concluded that these patients may benefit from reducing their SBP below 140 mm Hg [18]. Similar patterns were also observed in the PRavastatin Or atorVastatin Evaluation and Infection Therapy-Thrombolysis In Myocardial Infarction (PROVE IT-TIMI) 22 trial, which found that the lowest event rates were associated with an SBP range of 130–140 mm Hg in patients with acute coronary syndrome. The Comparison of Amlodipine vs Enalapril to Limit Occurrences of Thrombosis study conducted by Nissen et al. included 1991 CAD patients with an average baseline BP of 129/78 mm Hg and demonstrated that treatment with amlodipine resulted in reduced adverse cardiovascular events in normotensive patients with CAD [19]. The above studies suggest that the optimal SBP level in patients with CAD is clearly lower than 140 mm Hg and perhaps in 120 mm Hg range [20]. According to the recent findings of a network meta-analysis, the 2017 American College of Cardiology/American Heart Association practice guideline updated its recommendation for an SBP target to <130 mm Hg in adults with SIHD [8, 14, 21].

Because coronary perfusion occurs mainly during diastole, DBP has gradually become the focus of attention in hypertensive patients with CAD. A secondary analysis using data obtained from the INVEST observed a J-shaped association between BP and cardiovascular events, in which the J-curve was relatively more prominent in diastole than in systole. Moreover, the authors found that patients who had coronary revascularization seemed to have the potential to tolerate the lower DBP, compared with those who had not [13, 17]. The current BP management guideline recommends a target DBP of <80 mm Hg in patients with SIHD [8]. In addition, the PROVE IT-TIMI 22 trial suggested a DBP < 70 mm Hg may be dangerous in patients with high-risk unstable angina [22]. Furthermore, the Treating to New Targets Trial revealed that a low DBP of less than 60–70 mm Hg was accompanied by an increased risk of cardiovascular events in patients with CAD [23]. Similar findings were observed in the Atherosclerosis Risk in Communities study [24]. Therefore, DBP should be considered during hypertension treatment [25]. Unlike the secondary analysis of the previous studies, our results showed that a DBP around 65 mm Hg in patients with CAD was still safe and did not increase CVD events.

In addition, we found that participants with a history of coronary revascularization had lower DBP regardless of antihypertensive treatment, compared to those without such a history. The most probable explanation is that individuals with revascularization have worse atherosclerotic lesions and poorer arterial elasticity. The loss of arterial elasticity results in a decline in DBP and impairment of the auto-regulatory process of coronary circulation [26, 27]. Although revascularization opens culprit vessels and restores blood flow to ischemic areas, such a procedure per se cannot prevent atherosclerotic progression. On the one hand, it is not recommended to achieve an SBP target of 120 mm Hg in CAD patients with coronary revascularization, as our results suggest that this may increase the risk of stroke. On the other hand, excessive diastolic hypotension may have attenuated the benefits from intensive SBP treatment [28]. Further research is required to evaluate the minimum DBP target that has no effect on myocardial perfusion while ensuring adequate reduction in SBP in CAD patients with coronary revascularization.

We analyzed the baseline characteristics of participants with and without CAD or coronary revascularization. We observed that traditional cardiovascular risk factors, including heart rate, total cholesterol, low-density lipoprotein cholesterol, and use of aspirin and statins, were better controlled in participants who had CAD or had undergone coronary revascularization than in those who had not. In light of this, we postulate that the benefits of further intensive BP treatment might have been diluted in this population due to existing well-executed secondary prevention. It is, however, undeniable that the relatively limited subject number reduced the power of the study.

Several limitations of the present study should be noted. First, it was based on a post-hoc analysis of data obtained from a randomized controlled trial. The number of participants with CAD was relatively small, which may reduce the power of our statistical analysis. Second, the SPRINT study population excluded patients with a history of diabetes mellitus or stroke; thus, our study conclusions may not apply to other subsets of patients. Third, BP measurements in an unattended office are not an alternative to home BP measurements, given the low correlation and wide range of differences between the two groups [29]. Therefore, these results should be carefully interpreted and further validated through future studies.

In conclusion, the present study suggested that patients without CAD whose SBP is around 120 mm Hg have greater benefits for clinical outcomes, while this benefits might be attenuated in patients with CAD who are under better secondary prevention. The risk of stroke was increased by intensive BP treatment in patients with a history of coronary revascularization, though the confidence interval was wide. These findings may serve to inform current clinical practice and future trial design.

### Summary

#### What is known about the topic

- The prevalence of hypertension in individuals with pre-existing CAD ranges from 30 to 70%.
- Uncontrolled BP increased mortality in patients with CAD.
- Recent practice guidelines recommend a BP target of less than 130/80 mm Hg in individuals with SIHD.

#### What this study adds

- The optimal SBP level in hypertensive patients without CAD and diabetes mellitus is around 120 mm Hg.
- In non-diabetic hypertensive patients with CAD who are under better secondary prevention, the benefit from intensive BP treatment might be attenuated.
- The risk of stroke is increased by an SBP target of 120 mm Hg in patients with a history of coronary revascularization, though the confidence interval was wide.

## Supplementary information

Supplementary figure and table legends

Supplementary Figure 1

Supplementary Table 1

Supplementary Table 2

Supplementary Table 3

Supplementary Table 4
