# Supplementary material for: Intensive blood pressure treatment in coronary artery disease: implications from the Systolic Blood Pressure Intervention Trial (SPRINT)
Source: J Hum Hypertens. 2021 Feb 15;36(1):86–94. doi: 10.1038/s41371-021-00494-8 (PMC8766284; doi:10.1038/s41371-021-00494-8)
Supplement: Supplementary file 1 — Supplementary figure and table legends [file 41371_2021_494_MOESM1_ESM.doc]

**Supplementary figure and table legends**

**Supplementary Figure 1** Consort diagram leading to the subset of SPRINT participants in the current analyses. *CAD* coronary artery disease.

**Supplementary Table 1** Baseline characteristics of participants with and without CAD. Values are mean ± SD or number (%). *CAD* coronary artery disease, *LDL-C* Low-density lipoprotein cholesterol, *HDL-C* High-density lipoprotein cholesterol, *eGFR* estimated glomerular filtration rate.

**Supplementary Table 2** Baseline characteristics of CAD participants with and without coronary revascularization. Values are mean ± SD or number (%). *CAD* coronary artery disease, *LDL-C* Low-density lipoprotein cholesterol, *HDL-C* High-density lipoprotein cholesterol, *eGFR* estimated glomerular filtration rate.

**Supplementary Table 3** BP medications at last visit time of CAD participants by BP treatment arm. Values are number (%). 254 participants missing data at last visit time. *ACEI* angiotensin-converting enzyme inhibitors, *ARB* angiotensin II receptor blocker, *CCB* calcium channel blocker.

**Supplementary Table 4** BP medication changes of CAD participants with and without coronary revascularization. Values are number (%). 481 participants missing data at baseline, 223 participants missing data at last visit time. *ACEI* angiotensin-converting enzyme inhibitors, *ARB* angiotensin II receptor blocker, *CCB* calcium channel blocker.
