# Supplementary figures and images for: Intensive blood pressure treatment in coronary artery disease: implications from the Systolic Blood Pressure Intervention Trial (SPRINT)
Source: J Hum Hypertens. 2021 Feb 15;36(1):86–94. doi: 10.1038/s41371-021-00494-8 (PMC8766284; doi:10.1038/s41371-021-00494-8)

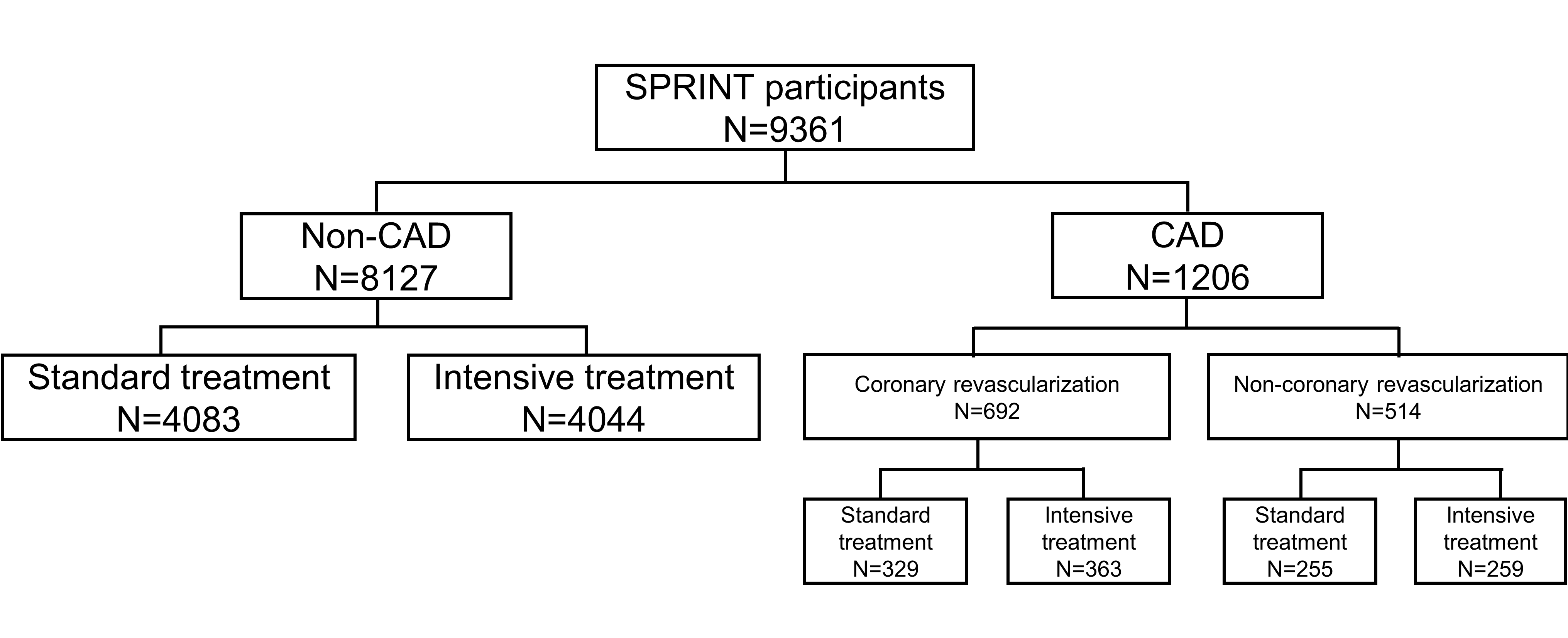

Supplement: Supplementary file 2 — Supplementary Figure 1 [file 41371_2021_494_MOESM2_ESM.tif]
