# Supplementary material for: Intensive blood pressure treatment in coronary artery disease: implications from the Systolic Blood Pressure Intervention Trial (SPRINT)
Source: J Hum Hypertens. 2021 Feb 15;36(1):86–94. doi: 10.1038/s41371-021-00494-8 (PMC8766284; doi:10.1038/s41371-021-00494-8)
Supplement: Supplementary file 3 — Supplementary Table 1 [file 41371_2021_494_MOESM3_ESM.docx]

**Supplementary Table 1** Baseline characteristics of participants with and without CAD

| Characteristics | Non-CAD | CAD | | *P* value |
| --- | --- | --- | --- | --- |
| N | 8127 | | 1206 |  |
| Age, years | 67.6±9.4 | | 69.9±9.1 | <0.001 |
| Female, n (%) | 3073 (37.8) | | 246 (20.4) | <0.001 |
| Black race, n (%) | 2714 (33.4) | | 220 (18.2) | <0.001 |
| Body mass index, kg/m2 | 29.9±5.8 | | 29.6±5.4 | 0.045 |
| Systolic blood pressure, mm Hg | 140.0.7±15.5 | | 137.8±15.8 | <0.001 |
| Diastolic blood pressure, mm Hg | 78.7±11.8 | | 74.2±12.1 | 0.054 |
| Heart rate, bpm | 66.8±11.6 | | 62.9±11.2 | <0.001 |
| Chronic kidney disease, n (%) | 2199 (27.1) | | 442 (36.7) | <0.001 |
| Smoking status, n (%) |  | |  | <0.001 |
| Never smoked | 3715 (45.8) | | 401 (33.3) |  |
| Former smoker | 3321 (40.9) | | 647 (53.6) |  |
| Current smoker | 1081 (13.3) | | 158 (13.1) |  |
| Total cholesterol, mg/dl | 193.6±40.2 | | 166.4±40.1 | <0.001 |
| LDL-C, mg/dl | 115.4±34.3 | | 92.3±33.7 | <0.001 |
| HDL-C, mg/dl | 53.5±14.7 | | 48.8±12.3 | <0.001 |
| Triglycerides, mg/dl | 125.7±91.5 | | 127.6±84.3 | 0.493 |
| Fasting plasma glucose, mg/dl | 98.6±13.5 | | 100.5±13.5 | <0.001 |
| eGFR, mL/min/1.73 m2 | 72.4±20.6 | | 67.4±19.8 | <0.001 |
| Creatinine, mg/dl | 1.1±0.3 | | 1.1±0.3 | <0.001 |
| Serum sodium, mmol/l | 140.1±2.4 | | 140.1±2.6 | 0.732 |
| Serum potassium, mmol/l | 4.2±0.4 | | 4.3±0.4 | <0.001 |
| Statin use, n (%) | 3101 (38.4) | | 946 (79.0) | <0.001 |
| Aspirin use, n (%) | 3722 (45.9) | | 1027 (85.2) | <0.001 |
| Antihypertensive agents, n (%) |  | |  | <0.001 |
| 1 | 2494 (30.7) | | 246 (20.4) |  |
| 2 | 2811 (34.6) | | 472 (39.1) |  |
| 3 | 1584 (19.5) | | 333 (27.6) |  |
| 4 | 398 (4.9) | | 97 (8.0) |  |

Values are mean ± SD or number (%).

*CAD* coronary artery disease, *LDL-C* Low-density lipoprotein cholesterol, *HDL-C* High-density lipoprotein cholesterol, *eGFR* estimated glomerular filtration rate.
