# Supplementary material for: Intensive blood pressure treatment in coronary artery disease: implications from the Systolic Blood Pressure Intervention Trial (SPRINT)
Source: J Hum Hypertens. 2021 Feb 15;36(1):86–94. doi: 10.1038/s41371-021-00494-8 (PMC8766284; doi:10.1038/s41371-021-00494-8)
Supplement: Supplementary file 4 — Supplementary Table 2 [file 41371_2021_494_MOESM4_ESM.doc]

**Supplementary Table 2** Baseline characteristics of CAD participants with and without coronary revascularization

| Characteristics | CAD | | *P* value |
| --- | --- | --- | --- |
|  | Non-coronary revascularization | Coronary revascularization |  |
| N | 514 | 692 |  |
| Age, years | 69.6±9.4 | 70.1±9.0 | 0.308 |
| Female, n (%) | 144 (28.0) | 102 (14.7) | <0.001 |
| Black race, n (%) | 136 (26.5) | 84 (12.1) | <0.001 |
| Body mass index, kg/m2 | 29.6±5.6 | 29.5±5.2 | 0.676 |
| Systolic blood pressure, mm Hg | 138.7±15.5 | 137.1±16.1 | 0.088 |
| Diastolic blood pressure, mm Hg | 74.9±12.2 | 73.6±11.9 | 0.054 |
| Heart rate, bpm | 64.1±11.8 | 61.9±10.6 | 0.001 |
| Chronic kidney disease, n (%) | 192 (37.4) | 250 (36.1) | 0.662 |
| Smoking status, n (%) |  |  | 0.353 |
| Never smoked | 177 (34.4) | 224 (32.4) |  |
| Former smoker | 264 (51.4) | 383 (55.3) |  |
| Current smoker | 73 (14.2) | 85 (12.3) |  |
| Total cholesterol, mg/dl | 167.4±40.0 | 165.5±40.2 | <0.001 |
| LDL-C, mg/dl | 95.5±35.1 | 90.0±32.5 | 0.005 |
| HDL-C, mg/dl | 50.1±13.0 | 47.8±11.6 | 0.001 |
| Triglycerides, mg/dl | 127.3±81.1 | 127.9±86.5 | 0.916 |
| Fasting plasma glucose, mg/dl | 99.8±15.1 | 101.1±12.2 | 0.097 |
| eGFR, mL/min/1.73 m2 | 67.5±21.3 | 67.4±18.7 | 0.916 |
| Creatinine, mg/dl | 1.2±0.4 | 1.1±0.3 | 0.619 |
| Serum sodium, mmol/l | 140.1±2.6 | 140.1±2.5 | 0.616 |
| Serum potassium, mmol/l | 4.3±0.4 | 4.3±0.4 | 0.006 |
| Statin use, n (%) | 377 (74.1) | 569 (82.6) | <0.001 |
| Aspirin use, n (%) | 412 (80.2) | 615 (88.9) | <0.001 |
| Antihypertensive agents, n (%) |  |  | 0.042 |
| 1 | 105 (20.4) | 141 (20.4) |  |
| 2 | 185 (36.0) | 287 (41.5) |  |
| 3 | 155 (30.2) | 178 (25.7) |  |
| 4 | 37 (7.2) | 60 (8.7) |  |

Values are mean ± SD or number (%).

*CAD* coronary artery disease, *LDL-C* Low-density lipoprotein cholesterol, *HDL-C* High-density lipoprotein cholesterol, *eGFR* estimated glomerular filtration rate.
