# Supplementary material for: Intensive blood pressure treatment in coronary artery disease: implications from the Systolic Blood Pressure Intervention Trial (SPRINT)
Source: J Hum Hypertens. 2021 Feb 15;36(1):86–94. doi: 10.1038/s41371-021-00494-8 (PMC8766284; doi:10.1038/s41371-021-00494-8)
Supplement: Supplementary file 5 — Supplementary Table 3 [file 41371_2021_494_MOESM5_ESM.docx]

**Supplementary Table 3** BP medications at last visit time of CAD participants by BP treatment arm.

| BP medications | Intensive BP treatment | Standard BP treatment | *P* value |
| --- | --- | --- | --- |
| N | 500 | 452 |  |
| ACEI | 188 (37.6) | 161 (35.6) | 0.53 |
| ARB | 193 (38.6) | 124 (27.4) | <0.001 |
| β-blocker | 352 (70.4) | 299 (66.2) | 0.16 |
| CCB | 267 (53.4) | 177 (39.2) | <0.001 |
| diuretics | 286 (57.2) | 201 (44.4) | <0.001 |
| α-blocker | 55 (11.0) | 21 (4.6) | <0.001 |

Values are number (%).

254 participants missing data at last visit time.

*ACEI* angiotensin-converting enzyme inhibitors, *ARB* angiotensin II receptor blocker, *CCB* calcium channel blocker.
