# Supplementary material for: Intensive blood pressure treatment in coronary artery disease: implications from the Systolic Blood Pressure Intervention Trial (SPRINT)
Source: J Hum Hypertens. 2021 Feb 15;36(1):86–94. doi: 10.1038/s41371-021-00494-8 (PMC8766284; doi:10.1038/s41371-021-00494-8)
Supplement: Supplementary file 6 — Supplementary Table 4 [file 41371_2021_494_MOESM6_ESM.docx]

**Supplementary Table 4** BP medication changes of CAD participants with and without coronary revascularization.

| Medications | Revascularization | Non-revascularization | *P* value |
| --- | --- | --- | --- |
| At baseline | N=420 | N=305 |  |
| ACEI | 51 (12.1) | 43 (14.1) | 0.44 |
| ARB | 12 (2.9) | 14 (4.6) | 0.22 |
| β-blocker | 64 (15.2) | 49 (16.1) | 0.76 |
| CCB | 40 (9.5) | 32 (10.5) | 0.67 |
| Diuretics | 41 (9.8) | 31 (10.2) | 0.86 |
| α-blockers | 12 (2.9) | 15 (4.9) | 0.15 |
| At last visit time | N=564 | N=419 |  |
| ACEI | 214 (61.3) | 135 (38.7) | 0.06 |
| ARB | 177 (55.8) | 140 (44.2) | 0.50 |
| β-blocker | 400 (61.4) | 251 (38.6) | <0.001 |
| CCB | 247 (55.6) | 197 (44.4) | 0.32 |
| Diuretics | 260 (53.4) | 227 (46.6) | 0.01 |
| α-blockers | 42 (55.3) | 34 (44.7) | 0.70 |

Values are number (%).

481 participants missing data at baseline, 223 participants missing data at last visit time.

*ACEI* angiotensin-converting enzyme inhibitors, *ARB* angiotensin II receptor blocker, *CCB* calcium channel blocker.
